# Antioxidant production promotes defense mechanism and different gene expression level in *Zea* *mays* under abiotic stress

**DOI:** 10.1038/s41598-024-57939-6

**Published:** 2024-03-26

**Authors:** Qurban Ali, Adnan Sami, Muhammad Zeshan Haider, Muhammad Ashfaq, Muhammad Arshad Javed

**Affiliations:** Department of Plant Breeding and Genetics, Faculty of Agriculture, University of the Punjab, Lahore, 54590 Pakistan

**Keywords:** Maize, Salt, Drought, Antioxidants, Root length, Shoot length, Plant sciences, Plant stress responses

## Abstract

The growth and productivity of maize are severely affected by soil salinity. The crucial determinants for the future performance of plants are productive for seed germination and seedling establishment; however, both stages are liable to soil salinity. For grain, maize is an economically significant crop sensitive to abiotic stresses. However, little is known about defense responses by the salinity-induced antioxidant and oxidative stress in maize. In our work, the commercially available maize variety Raka-Poshi was grown in pots for 30 days under greenhouse conditions. To evaluate the salt-induced oxidative/antioxidant responses in maize for salt stress 0, 25, 50, 75, 100 and 150 mM concentrations, treatments were provided using sodium chloride (NaCl). All the biochemical indices were calculated under all NaCl concentrations, while drought was induced by up to 50% irrigation water. After 30 days of seed germination, the maize leaves were collected for the measurement of lipid peroxidase or malondialdehyde (MDA), glutathione reductase (GR), guaiacol peroxidase (GPOD), hydrogen peroxide (H_2_O_2_), superoxide dismutase (SOD), lipoxygenase (LOX), catalase (CAT), ascorbate peroxidase (APOD) and glutathione-*S*-transferase (GST). The results revealed a 47% reduction under 150 mM NaCl and 50% drought stress conditions. The results have shown that the successive increase of NaCl concentrations and drought caused an increase in catalase production. With successive increase in NaCl concentration and drought stress, lower levels of H_2_O_2_, SOD, and MDA were detected in maize leaves. The results regarding the morphology of maize seedlings indicated a successive reduction in the root length and shoot length under applications of salt and drought stress, while root-to-shoot weights were found to be increased under drought stress and decreased under salt stress conditions During gene expression analysis collectively indicate that, under drought stress conditions, the expression levels of all nine mentioned enzyme-related genes were consistently downregulated.

## Introduction

The *Zea* *mays* is the most important cereal crop used as food and feed, and its raw materials are used in various critical industrial by-products. Maize has a significant position among existing plant cropping systems for Pakistan maize growing areas. Maize ranks third after the rice and wheat crops for grain yield and production in the country. It is produced in almost all provinces of the country, where Punjab and KPK are Pakistan's main maize production and productivity areas. The estimate for yield in Pakistan is about 70% of production is used indirectly or directly in food, while the rest of its production is used for starch formation and poultry industries for feed formation. Maize grain was constituted about grain protein, grain oil, grain crude fibre, grain starch, the embryo as 9.7396%, 4.85%, 9.4392%, 71.966%, 11.77%, respectively, while the fodder contains acid detergent fibre, nutrient detergent fibre, fodder cellulose, fodder dry matter, fodder crude protein, fodder moisture as 22.988%, 51.696%, 28.797%, 40.178%, 26.845%, 10.353%, 9.095% respectively^1,2^. In Pakistan, maize is grown or cultivated two times a year, i.e., in the country's autumn and spring seasons.

Crop protection and its management are important in improving grain yield and production under every environmental condition^3^. The management inputs include improved seed varieties, irrigation, planting patterns, crop sowing times, the use of fertilizers, and crop plant population, which play an influential and significant role in enhancing crop plant and grain yield under any environmental condition. The maize crop plant is generally cultivated or grown under the irrigated field conditions of Pakistan^4^. The water has been shortening due to shortage or less rainfalls; the water has been becoming scarce throughout the country, causing salt stress^5,6^. The water limitations and salt stresses also adversely affect other crop plants like wheat, rice, barley, and oat crops^7^. The maize plant suffers from salt and drought stress at anthesis and the grain filling stages up to 40–80% yield loss. Salt has been considered one of the major factors affecting plant growth and the grain yield of maize. There has been a need to recognize a suitable and efficient technique for maize cultivation that can resist salt and drought stress in environmental conditions^8^. The maize has higher water demands, which can give higher grain production even when the water, mineral, and other soil nutrients have become sufficient in amount and avail to plants quickly; the maize plant is also susceptible to salt and water deficit of moisture stress environment^9^ along with other stress environments like cold, heat, salt and alkaline conditions at anthesis period of plants^10^. The maize plant is a highly drought-stress-sensitive crop plant that is positively affected by drought stress at anthesis, pollination stage, and silk emergence. The requirements of water for maize crop plant are up to 500–800 mm for plant life cycle about 80 up to 110 days of crop growing^11^.

## Materials and methods

It has been confirmed that the experimental data collection complied with relevant institutional, national, and international guidelines and legislation with appropriate permissions from the authorities of the Department of Plant Breeding and Genetics, University of the Punjab, Lahore, Pakistan.

The maize seeds of cultivated variety Raka-Poshi were collected from the local seed market of Lahore. The seeds of maize were sown in pots [8 cm (diameter) × 12 cm (depth)] containing 800 g of sterilized pure sand; the experiment was replicated thrice times under factorial design following Randomized Complete Block Design. The salt treatment was provided in the pure sand culture before seed sowing by adding 300 ml each of 0, 25, 50, 100, and 150 mM NaCl solution. Total amount of irrigation for normal conditions was applied as 200 ml water, while 80% irrigation (160 ml water), 60% irrigation (120 ml water), 40% irrigation (80 ml water) and 20% irrigation (40 ml water). The drought condition was indued while reducing the overall percentage of irrigation application. Plants were grown to develop for 30 days and weekly for salt and drought stress treatments. The pots were kept in the greenhouse under natural conditions [with photo-synthetically active radiation range of about 690–730 µmole m^−2^ s^−1^ under sunlight while on cloudy day 400 µmol m^−2^ s^−1^ photo-synthetically active radiation (Spectrum Technologies, Inc., IL, USA). The 27 ± 2 °C temperature, 75% relative humidity, and 11 h photoperiod were observed during the whole experiment. The maize plants were uprooted and washed the roots with deionized water. On day 30th, leaf samples were collected and processed for enzymes, H_2_O_2_, and lipid peroxidation analysis using different biochemical analyses described below. The morphological traits were recorded for salt and drought stress conditions, including root length, shoot length, root weight, and root-to-shoot length ratio.

### Lipid peroxidation

To assess lipid peroxidation and quantify malondialdehyde (MDA), we employed a modified version of the thiobarbituric acid reactive substance (TBARS) method as outlined by Heath and Packer (1968)^12,13^. The leaf samples of 0.25 g were centrifuged at 12,000 rpm for 30 min and homogenized using the 0.1% trichloroacetic acid (TCA) solution. The supernatant obtained was incubated for 30 min at 95 °C with a ratio of 20% TCA containing 0.5% thiobarbituric acid (TBA). For 10 min through cooling of the test tubes in the ice bath to stop the reaction, the product of stopped reactions was centrifuged at 10,000 rpm up to 15 min. The 532 nm absorbance for supernatant was observed while the non-specific light absorption of 600 nm was subtracted from total observed values from the standard extinction coefficient 155 mM^−1^ cm^−1^; hence, the expressed value of MDA was calculated in nmol g^−1^ Fwt of leaves.

## H_2_O_2_ calculation

The leaf samples (0.25 g) were obtained and centrifuged at 14,000 rpm at 4 °C for 15 min while homogenized using 0.1% trichloroacetic acid. Before the measurement of the oxidation product on A390, a supernatant of 0.3 ml was mixed with 1 ml of 1 M potassium iodide solution and 1.7 m of potassium phosphate buffer with pH 7.0 and nursed for 5 min. From calculating the standard curves of H_2_O_2_ concentration, which was considered and prepared from the known concentrations of H_2_O_2_ and expressed in nmol g^−1^ Fwt^14,15^.

## Enzyme assays

The pre-chilled mortar and pestle leaf tasters (0.25 g) homogenized in 100 mM of potassium phosphate as a buffer of pH 7.0, inclosing 0.1 mM phenylmethylsulphonyl fluoride (PMSF), 0.5 mM EDTA and 2% PVP. At 4 °C, the extraction buffer was also confined 5 mM ascorbate, homogenated, and then centrifuged at 14,000 rpm for 30 min. The catalase spectrophoto metric test assesses the preservation of stable electrophoretic mobility of catalase by observing its behavior in the presence of dithiothreitol (DTT)^16^. The specific CAT activity was articulated as µmol min^−1^ mg^−1^ protein. SOD activity was determined by the method of Dhindsa^17,18^ as in the absence of an enzyme, one unit of SOD activity was defined as a 50% inhibition of the initial rate of the reaction caused by enzymatic activity^19,20^. The SOD-specific activity was expressed as unit’s min^−1^ mg^−1^ protein.

The GPOD activity was analyzed through a reaction mixture containing 10 mM H_2_O_2_, 50 mM potassium phosphate buffer with pH 7.0, 0.05% guaiacol, and enzyme. The specific GPOD activity was expressed as µmole min^−1^ mg^−1^ protein. The APOD action was examined in a reaction mixture having 50 mM potassium phosphate buffer (pH 7.0), 0.1 mM EDTA, 1.0 mM H_2_O_2_, 0.25 Mm ascorbic acid and catalyst. APOD-specific movement was shown as µmole of ascorbate oxidized min^−1^ mg^−1^ protein. GR activity was evaluated in a reaction mixture having 3 mM DNTB (5,5-dithio-bis-2-nitrobenzoic acid), 50 mM potassium phosphate buffer (pH 7.5), 0.1 mM EDTA, 2 mM NADPH and enzyme. The specific activity of GR was expressed as nmol min^−1^ mg^−1^ protein. The Bradford (1976) method determined protein content using BSA as the standard^21,22^. The 1-chloro-2,4-dinitrobenzene was used as a substrate for determining GST activity^23^.

### Statistical analysis

The randomized complete block design with two independent experiments, each in triplicate, was conducted to assess the antioxidant production against salt and drought stress conditions. The pooled or combined ANOVA for both experiments was calculated (Supplementary Materials Table 1) to assess the significance of results. The biochemical tests were carried out three times each for selected seedlings of maize. The analysis of variance was performed to find out the significance of results^24^ along with the least significant difference (LSD) at P < 0.05, and the standard error of the mean was also calculated. Principal component analysis was calculated to assess the variation among the studies traits. Broad sense heritability and genetic advance were recorded to assess the genetic behavior of studied traits.

### Gene expression data

To investigate the effect of drought stress on maize, two distinct maize lines W9706 (tolerant) and B73 susceptible, were selected to analyze their response to water shortage based on their genetic constitution. RNA seq data procured from NCBI GEO database (https://www.ncbi.nlm.nih.gov/geo/) (GSE223667) aiming to dig deeper into the expression profile of various gene families that regulate different enzymes in maize during dry spells. The Maize Genome map (*Zea* *mays* RefGen_V4) was also sourced from Phytozome v13's site: (https://phytozome-next.jgi.doe.gov/info/Zmays_RefGen_V4).

## Results

### Seed germination (%)

The results in Table 1 indicated the adverse effect of salt and drought stress on maize seed germination. The germination was decreased while increasing the salt and drought treatment concentration. Under control, germination was recorded as 98.28 ± 1.2391%, while under application of 80% irrigation (20% drought), germination was reported as 82.43 ± 1.0923%. The lowest seed germination, 32.98 ± 0.98% was recorded under 150 mM NaCl, while the minimum under 20% irrigation (80% drought) seed germination was 53.53 ± 0.2044%. The lowest seed germination under salt stress conditions was found under salt stress conditions, indicating that salt stress was more damaging than drought.

**Table 1:** The effects of varying NaCl concentrations and irrigation levels on key physiological parameters in maize seedlings, including seed germination, H_2_O_2_ content, lipid peroxidation, superoxide dismutase activity, and lipoxygenase content (nmol mg^−1^ Fwt.).

Treatments	Seed germination %	H_2_O_2_	Lipid peroxidation	Superoxide Dismutase	Lipoxygenase
Control	98.28 ± 1.2391a	23.10 ± 1.092f	2.314 ± 0.023f	1.231 ± 0.0023f	12.312 ± 0.536f
Salt stress					
25 mM NaCl	72.13 ± 1.0923b	102.133 ± 1.0237e	4.235 ± 0.6712e	2.023 ± 0.0289e	23.382 ± 0.1029e
50 mM NaCl	63.02 ± 1.2035c	130.158 ± 2.3011d	6.455 ± 0.2011d	3.054 ± 0.0012d	36.232 ± 0.1192d
75 mM NaCl	52.06 ± 1.0023d	143.131 ± 1.029c	9.947 ± 0.0526c	4.102 ± 0.022c	42.767 ± 0.326c
100 mM NaCl	43.77 ± 0.7824e	189.285 ± 3.124b	11.245 ± 0.6872b	5.0283 ± 0.011b	57.231 ± 1.027b
150 mM NaCl	32.98 ± 0.9823f	232.01 ± 2.0917a	16.927 ± 0.0236a	7.135 ± 0.0239a	68.984 ± 1.208a
Drought stress					
80% irrigation	82.43 ± 1.0923a	12.241 ± 0.734d	3.552 ± 0.7821d	2.671 ± 0.027d	15.242 ± 0.972d
60% irrigation	73 ± 0.9032b	104.472 ± 1.0463c	6.241 ± 0.2536c	3.917 ± 0.0182c	19.782 ± 0.1782c
40% irrigation	61.23 ± 1.1203c	150.24 ± 3.082b	9.278 ± 0.4621b	5.0172 ± 0.1294b	38.701 ± 1.238b
20% irrigation	53.53 ± 0.2044d	246.324 ± 2.1841a	17.234 ± 0.2674a	6.236 ± 0.2191a	46.592 ± 1.043a

Treatment groups include control, salt stress (25 mM to 150 mM NaCl), and drought stress (80% to 20% irrigation). Significantly different groups are denoted by distinct lowercase letters (a–f).

### Hydrogen peroxide (H_2_O_2_)

The hydrogen peroxide produced in maize seedlings under stress conditions during metabolism and photosynthesis due to the formation of ROS. The results (Table 1) showed that the higher H_2_O_2_ was under higher salt stress 150 mM NaCl as 232.01 ± 2.0917. Followed by 100 mM NaCl (189.285 ± 3.124) and 75 mM NaCl (143.131 ± 1.029 ) while under drought stress, the highest H_2_O_2_ was recorded under the application of only 20% irrigation (80% drought) as 246.324 ± 2.1841. and under 40% irrigation (150.24 ± 3.082). The lowest H_2_O_2_ was recorded under control conditions (23.10 ± 1.092 ) and 80% irrigation treatment (12.241 ± 0.734).

### Lipid peroxidation/malondialdehyde (MDA)

The lipid peroxidation was increased in maize seedlings under stress conditions during metabolism and photosynthesis due to the formation of ROS. The results (Table 1) showed that the higher lipid peroxidation was while under higher salt stress 150 mM NaCl as 16.927 ± 2.0917. Followed by 100 mM NaCl (11.245 ± 0.6872 ) and 75 mM NaCl (9.947 ± 0.0526) while under drought stress, the highest lipid peroxidation was recorded under the application of only 20% irrigation (80% drought) as 17.234 ± 0.2674. and under 40% irrigation (9.278 ± 0.4621). The lowest lipid peroxidation was recorded under control conditions (2.314 ± 0.023) and 80% irrigation treatment (3.552 ± 0.7821).

### Superoxide dismutase

The superoxide dismutase release was increased in maize seedlings under stress conditions during metabolism and photosynthesis in response to ROS production. The results (Table 1) showed that the higher superoxide dismutase was under higher salt stress 150 mM NaCl as 7.135 ± 0.0239 unit min^−1^ mg^−1^ protein. Followed by 100 mM NaCl (5.0283 ± 0.011) and 75 mM NaCl (4.102 ± 0.022) while under drought stress, the highest superoxide dismutase was recorded under the application of only 20% irrigation (80% drought) as 6.236 ± 0.2191 and under 40% irrigation (5.0172 ± 0.1294). The lowest superoxide dismutase was recorded under control conditions (1.231 ± 0.0023) and 80% irrigation treatment (2.671 ± 0.027).

### Lipoxygenase

The lipoxygenase formation was increased in maize seedlings under stress conditions during metabolism and photosynthesis due to the formation of ROS (reactive oxygen species). The results (Table 1) showed that the higher lipoxygenase was while under higher salt stress 150 mM NaCl as 68.984 ± 1.208. Followed by 100 mM NaCl (57.231 ± 1.027 ) and 75 mM NaCl (42.767 ± 0.326) while under drought stress, the highest lipoxygenase was recorded under the application of only 20% irrigation (80% drought) as 46.592 ± 1.043. and under 40% irrigation (38.701 ± 1.238 ). The lowest lipoxygenase was recorded under control conditions (12.312 ± 0.536) and 80% irrigation treatment (15.242 ± 0.972).

### Glutathione-*S*-transferase

The glutathione-*S*-transferase production was enhanced in maize seedlings under stress conditions during metabolism and photosynthesis in response to ROS production. The results (Table 2) showed that the higher glutathione-*S*-transferase was while under higher salt stress 150 mM NaCl as 51.227 ± 1.392. Followed by 100 mM NaCl (46.232 ± 1.220) and 75 mM NaCl (41.347 ± 1.203) while under drought stress, the highest glutathione-*S*-transferase was recorded under the application of only 20% irrigation (80% drought) as 43.201 ± 1.220 and under 40% irrigation (32.239 ± 1.002). The lowest glutathione-*S*-transferase was recorded under control conditions (18.152 ± 1.028) and 80% irrigation treatment (21.299 ± 1.022).

**Table 2:** Enzyme activity levels (nmol min^−1^ mg^−1^ protein) in maize seedlings subjected to varying NaCl concentrations and irrigation levels.

Treatments	Catalase	Guaiacol peroxidase	Ascorbate peroxidase	Glutathione reductase	Glutathione-*S*-transferase
Control	0.026 ± 0.0001f	0.057 ± 0.002f	0.235 ± 0.021f	7.241 ± 0.627f	18.152 ± 1.028f
Salt stress					
25 mM NaCl	0.038 ± 0.0002e	0.048 ± 0.001e	0.288 ± 0.012e	12.401 ± 0.348e	28.123 ± 1.230e
50 mM NaCl	0.045 ± 0.0001d	0.103 ± 0.003d	0.342 ± 0.011d	17.263 ± 0.221d	36.572 ± 1.053d
75 mM NaCl	0.058 ± 0.0002c	0.178 ± 0.004c	0.383 ± 0.022c	25.124 ± 0.212c	41.347 ± 1.203c
100 mM NaCl	0.089 ± 0.0001b	0.198 ± 0.003b	0.432 ± 0.001b	31.231 ± 1.023b	46.232 ± 1.220b
150 mM NaCl	0.098 ± 0.0002a	0.202 ± 0.002a	0.577 ± 0.031a	47.121 ± 1.002a	51.227 ± 1.392a
Drought stress					
80% irrigation	0.023 ± 0.0001d	0.045 ± 0.001d	0.254 ± 0.029d	15.273 ± 0.967d	21.299 ± 1.022d
60% irrigation	0.067 ± 0.0002c	0.098 ± 0.002c	0.387 ± 0.072c	19.223 ± 0.789c	28.231 ± 1.079c
40% irrigation	0.072 ± 0.0001b	0.169 ± 0.001b	0.582 ± 0.028b	28.256 ± 1.072b	32.239 ± 1.002b
20% irrigation	0.099 ± 0.0002a	0.253 ± 0.002a	0.612 ± 0.021a	39.282 ± 1.291a	43.201 ± 1.220a

Treatment categories include control, salt stress (25 mM to 150 mM NaCl), and drought stress (80% to 20% irrigation). Distinct lowercase letters (a–d) indicate statistically significant differences between treatment means. Enzymes assessed include catalase, guaiacol peroxidase, ascorbate peroxidase, glutathione reductase, and glutathione-*S*-transferase (nmol min^−1^ mg^−1^ protein).

### Glutathione reductase

The glutathione reductase production was enhanced in maize seedlings under stress conditions during metabolism and photosynthesis in response to ROS production. The results (Table 2) showed that the higher glutathione reductase was while under higher salt stress 150 mM NaCl as 47.121 ± 1.002. Followed by 100 mM NaCl (31.231 ± 1.023) and 75 mM NaCl (25.124 ± 0.212 ) while under drought stress, the highest glutathione reductase was recorded under the application. This of only 20% irrigation (80% drought) as 39.282 ± 1.291this and under 40% irrigation (28.256 ± 1.072). The lowest glutathione reductase was recorded under control conditions (7.241 ± 0.627) and 80% irrigation treatment (15.273 ± 0.967).

### Ascorbate peroxidase

The ascorbate peroxidase production was enhanced in maize seedlings under stress conditions during metabolism and photosynthesis in response to ROS production. The results (Table 2) showed that the higher ascorbate peroxidase was while under higher salt stress 150 mM NaCl as 0.577 ± 0.031. Followed by 100 mM NaCl (0.432 ± 0.001) and 75 mM NaCl (0.383 ± 0.022), while the highest ascorbate peroxidase was recorded under drought stress. This applies only to 20% irrigation (80% drought) as 0.612 ± 0.021and under 40% irrigation (0.582 ± 0.028). The lowest ascorbate peroxidase was recorded under control conditions (0.235 ± 0.021) and 80% irrigation treatment (0.254 ± 0.029).

### Guaiacol peroxidase

The guaiacol peroxidase production was enhanced in maize seedlings under stress conditions during metabolism and photosynthesis in response to ROS production. The results (Table 2) showed that the higher guaiacol peroxidase was under higher salt stress 150 mM NaCl as 0.202 ± 0.002. This was followed by 100 mM NaCl (0.198 ± 0.003) and 75 mM NaCl (0.178 ± 0.004) while under drought stress, the highest guaiacol peroxidase was recorded as under the application of only 20% irrigation (80% drought) as 0.253 ± 0.002 and under 40% irrigation (0.169 ± 0.001). The lowest guaiacol peroxidase was recorded under control conditions (0.057 ± 0.002) and 80% irrigation treatment (0.045 ± 0.001).

### Catalase

The catalase release was increased in maize seedlings under stress conditions during metabolism and photosynthesis in response to ROS production. The results (Table 2) showed that the higher catalase was, while under salt stress, 150 mM NaCl as 0.098 ± 0.0002. Followed by 100 mM NaCl (5.089 ± 0.0001) and 75 mM NaCl (0.058 ± 0.0002) while under drought stress, the highest catalase was recorded under the application of only 20% irrigation (80% drought) as 0.099 ± 0.0002 and under 40% irrigation (0.072 ± 0.0001). The lowest catalase was recorded under control conditions (0.026 ± 0.0001) and 80% irrigation treatment (0.023 ± 0.0001) (Table 2).

The root-to-shoot length ratio was recorded higher under the application of 25 mM NaCl (0.432) and 80% irrigation (1.50) while lower for 150 mM NaCl (0.282) and 1.050 (20% irrigation). The higher root-to-shoot length ratio indicated the tolerance of maize seedlings against salt and drought stress conditions. The higher leaf area of maize seedlings was reported under 25 mM NaCl (4.93 cm^2^) while under 60% irrigation (5.580 cm^2^). The lower leaf area was found under salt stress 3.656 cm^2^ (100 mM NaCl) and 3.73 cm^2^ (20% irrigation). The shoot and root length showed adverse effects of drought and salt stress on maize seedlings. The results showed that the leaf area decreased gradually with increased stress application due to increased drought and salt stress. The decrease in the leaf area indicated a decrease in the photosynthetic rate, which may reduce the production and potential of maize genotypes under stressful environmental conditions. The more extensive shoot and root length of maize seedlings were reported under 50 mM NaCl (8.6 cm, 13.31 cm) and 80% irrigation (9.3 cm, 13.50 cm). The lower shoot and root length under salt stress 7.1 cm and 12.1 cm (150 mM NaCl) and 2.32 cm 13.1 cm (20% irrigation), respectively (Table 3). The shoot and root length showed adverse effects of drought and salt stress on maize seedlings.

**Table 3:** A detailed account of morphological characteristics in maize seedlings under varying NaCl concentrations and irrigation levels.

Treatments	No. of Roots	Shoot length	leaf length	leaf width	Root length	Root weight	shoot weight	Leaf area	Root to shoot length ratio
Control	6.23c	7.3c	2.7b	2.2a	4a	0.084b	0.191c	4.396b	0.548a
Salt stress									
25 mM NaCl	7.23b	8.1b	2.9a	2.3a	13.15b	0.082c	0.181d	4.936a	0.432ab
50 mM NaCl	5.74d	8.6a	2.8a	2.21b	13.31c	0.080c	0.181d	4.351c	0.360b
75 mM NaCl	6.26c	7.9b	2.7b	2b	13.1c	0.088a	0.194b	3.996d	0.380b
100 mM NaCl	4.41c	7.7b	2.6c	1.9c	12.8d	0.084b	0.191c	3.656e	0.364b
150 mM NaCl	9.2a	7.1c	2.5c	2.2a	12.1e	0.080c	0.195a	4.070c	0.282c
Drought stress									
80% irrigation	10.24b	9.3a	2.92b	2.6a	13.5a	0.309a	0.395a	5.387b	1.500c
60% irrigation	11.12a	8.6b	2.9a	2.6a	13.4b	0.301b	0.322b	5.580a	1.375c
40% irrigation	9.45c	4.34c	2.7c	2.4b	13.1c	0.302b	0.312c	4.795c	1.250b
20% irrigation	10.28b	2.32d	2.4d	2.1c	13.1c	0.309a	0.399a	3.730d	1.050a

Parameters assessed include the number of roots, shoot length (cm), leaf length (cm), leaf width (cm), root length (cm), root weight (g), shoot weight (g), leaf area (cm^2^), and root-to-shoot length ratio. Distinct lowercase letters (a–e) denote significant differences between treatment means. Units for each parameter are presented where applicable.

The salt and drought stress caused injury in plant cells, leading to seedlings' death. It was found from the results that a higher number of roots per plant was reported under higher salt stress conditions 75 mM NaCl (9.2) followed by 25 mM NaCl (7.23), while it was lower under 100 mM NaCl (4.41) applications. Under drought stress conditions, a higher number of roots per plant was found for 60% irrigation (11.12), followed by 20% irrigation (10.28). The overall performance of maize seedlings was better under drought-stress conditions. With the increase in drought stress, the number of roots per plant decreased with increased salt and drought stress.

**Figure 1:**
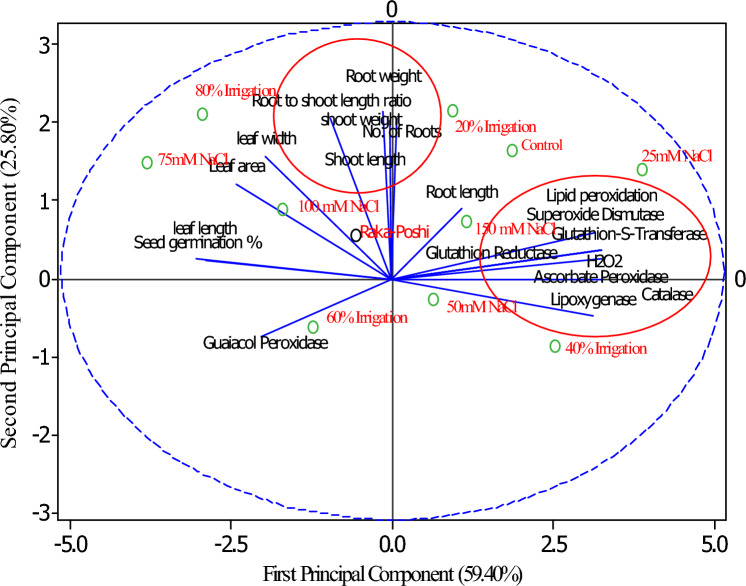
Principal component biplot for morphological and enzyme produced in maize under salt and drought stress conditions.

The results from Fig. 1 revealed that the performance of maize seedlings under drought and salt stress conditions was better for antioxidant enzymes produced by seedlings. Around all of the enzymes were found strongly corrected with each other for their release under a stressful environment. The selection of maize genotypes and hybrids may help develop stress tolerance and stress-resistant crop plant varieties.

It was persuaded from results given in Tables 4 and 5 showed significant differences among the treatments used as drought and salt stress. The results from Table 4 indicated that higher broad sense heritability was recorded for most of the studied traits expect glutathione-s-transferase, glutathione reductase, root weight, shoot weight and ascorbate peroxidase under drought stress condition. The highest genetic advance was recorded for root length, seed germination %age, shoot length, superoxide dismutase, guaiacol peroxidase, H_2_O_2_, Guaiacol peroxidase, MDA and lipoxygenase. The results from Table 5 indicated that higher broad sense heritability was recorded for most of the studied traits expect glutathione reductase, root weight and shoot weight under salt stress conditions. The highest genetic advance was recorded for root length, seed germination %age, shoot length, superoxide dismutase, H_2_O_2_, MDA and lipoxygenase.

**Table 4:** Genetic components for various traits of maize under drought stress conditions.

Traits	M.S	G.M	GV	GCV %	PV	PCV %	EV	ECV %	h^2^bs%	GA%
Root length	17.288*	12.593	5.762	67.642	5.764	67.656	0.002	1.381	99.958	89.610
Root weight	0.012*	0.319	0.003	9.911	0.006	13.289	0.003	8.853	55.621	13.129
Root-to-shoot length ratio	0.175*	1.268	0.058	21.301	0.060	21.777	0.003	4.528	95.676	28.219
Seed germination %age	553.422*	69.943	184.473	162.403	184.475	162.404	0.002	0.535	99.999	215.146
shoot length	25.348*	6.261	8.448	116.162	8.451	116.183	0.003	2.189	99.965	153.887
SOD	7.458*	4.243	2.485	76.532	2.488	76.570	0.003	2.427	99.900	101.386
Leaf length	0.105*	2.759	0.034	11.139	0.037	11.523	0.002	2.949	93.449	14.757
Leaf width	0.110*	2.439	0.036	12.132	0.038	12.498	0.002	3.003	94.226	16.072
Shoot weight	0.016*	0.376	0.004	10.899	0.007	13.415	0.002	7.821	66.010	14.439
catalase	0.003*	0.094	0.001	9.312	0.001	9.868	0.000	3.265	89.051	12.336
Gultathione-*S*-transferase	0.008*	0.078	0.002	14.879	0.004	23.004	0.002	17.544	41.834	19.711
Glutathione reductase	0.001*	0.053	0.000	6.194	0.001	12.311	0.001	10.640	25.311	8.205
Guaiacol peroxidase	0.030*	0.048	0.010	45.491	0.010	46.398	0.000	9.129	96.129	60.265
Ascorbate peroxidase	0.019*	0.067	0.005	28.293	0.008	34.062	0.002	18.966	68.997	37.482
H_2_O_2_	23,560.600*	132.230	7853.532	770.668	7853.536	770.669	0.003	0.509	99.970	1020.954
Leaf area	1.508*	4.868	0.502	32.107	0.504	32.184	0.002	2.225	99.522	42.535
MDA	88.700*	8.586	29.553	185.525	29.595	185.657	0.042	6.994	99.858	245.777
Lipoxygenase	569.428*	28.745	189.809	2.570	189.811	0.479	0.002	0.834	99.999	340.420
No of roots	4.318*	9.993	1.438	0.379	1.441	0.120	0.002	1.550	99.833	50.262

*Significant at 5% probability level, mean sum of squares (M.S), grand mean (G.M), genotypic variance (GV), genotypic coefficient of variance (GCV %), phenotypic variance (PV), phenotypic coefficient of variance (PCV %), environmental variance (EV), environmental coefficient of variance (ECV %), broad sense heritability (h^2^bs %), genetic advance (GA).

**Table 5:** Genetic components for various traits of maize under salt stress conditions.

Traits	M.S	G.M	GV	GCV %	PV	PCV %	EV	ECV %	h^2^bs%	GA%
Root length	13.7301*	12.355	4.577	60.863	4.577	60.864	0.0001	0.284	99.998	80.629
Root weight	0.0003*	0.102	0.000	1.808	0.000	4.783	0.0002	4.428	14.286	2.395
Root-to-shoot length ratio	0.0127*	0.394	0.004	10.361	0.004	10.374	0.0000	0.504	99.764	13.727
Seed germination %age	916.763*	55.656	305.588	234.321	305.588	234.321	0.0001	0.134	99.99	310.421
shoot length	0.781*	7.863	0.260	18.193	0.260	18.200	0.0002	0.504	99.923	24.102
SOD	10.742*	4.098	3.581	93.474	3.581	93.477	0.0002	0.699	99.994	123.831
Leaf length	0.060*	2.719	0.019	8.403	0.022	8.913	0.0024	2.971	88.889	11.132
Leaf width	0.0668*	2.146	0.022	10.170	0.022	10.218	0.0002	0.989	99.063	13.473
Shoot weight	0.0004*	0.207	0.000	2.198	0.000	3.108	0.0001	2.198	50.000	2.912
Catalase	0.0028*	0.098	0.001	9.512	0.001	10.034	0.0001	3.194	89.865	12.601
Gultathione-*S*-transferase	0.0004*	0.059	0.00001	4.694	0.0001	4.871	0.0001	1.302	92.857	6.218
Glutathione reductase	0.0013*	0.084	0.00001	7.015	0.001	7.817	0.0001	3.450	80.519	9.293
Guaiacol peroxidase	0.0015*	0.075	0.00001	7.746	0.001	8.563	0.0001	3.651	81.818	10.262
Ascorbate peroxidase	0.0016*	0.076	0.00001	8.002	0.001	8.786	0.0001	3.627	82.955	10.601
H_2_O_2_	9432.470*	150.847	3144.157	456.545	3144.157	456.545	0.0002	0.115	99.99	604.815
Leaf area	0.558*	4.233	0.186	20.962	0.186	20.962	0.0001	0.154	99.995	27.769
MDA	66.278*	9.315	22.093	154.004	22.093	154.004	0.0001	0.104	99.99	204.019
Lipoxygenase	948.847*	43.650	316.282	2.692	316.282	0.407	0.0001	0.151	99.99	356.602
No of roots	7.701*	6.566	2.567	0.625	2.567	0.244	0.0001	0.123	99.99	82.833

*Significant at 5% probability level, mean sum of squares (M.S), grand mean (G.M), genotypic variance (GV), genotypic coefficient of variance (GCV %), phenotypic variance (PV), phenotypic coefficient of variance (PCV %), environmental variance (EV), environmental coefficient of variance (ECV %), broad sense heritability (h^2^bs %), genetic advance.

The drought response in different maize strains, particularly how specific genes regulate hydrogen peroxide (H_2_O_2_) enzyme activity. The primary focus was on a key gene known as peroxidase 4-like [*Momordica* *charantia*]^25^. By comparing transcriptomes of W9706 (drought-tolerant line) and B73 (drought-susceptible line) using GSE223667 RNA seq data. Findings indicated that all genes tied to activates of the enzyme mentioned above generally experienced reduced expression under drought conditions; more specifically, six precise genes (Zm00001d022457, Zm00001d037550, Zm00001d040702, Zm00001d009140, Zm00001d010925 and Zm00001d009373) showed significant down-regulated during drought stress. For the expression of Ascorbate Peroxidase enzyme, mainly focusing on the cordate peroxidase 1 gene from Arabidopsis thaliana^26^. More notably, an observation in two distinct genes (Zm00001d007569, Zm00001d016802) was significantly down-regulated, which presented substantial decreases during periods lacking sufficient water reserves. Guaiacol peroxidase enzyme action was also studied under the same conditions. The transcriptome of its crucial gene peroxidase PPOD1-like precursor [*Hydra* *vulgaris*] was examined^27,28^.

**Figure 2:**
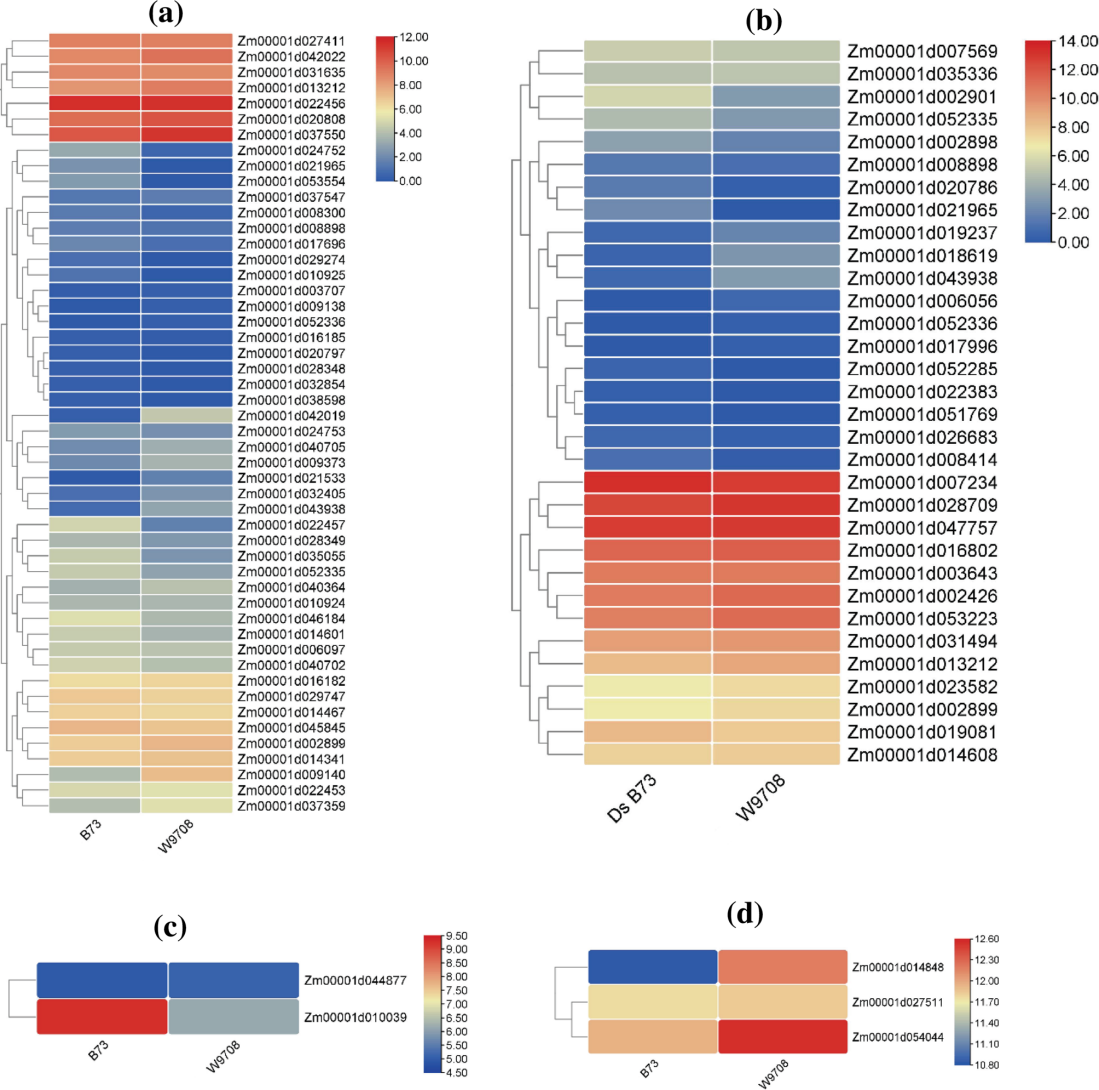
Gene expression level of (**a**) hydrogen peroxide (peroxidase 4-like [*Momordica* *charantia*]), (**b**) ascorbate peroxidase (ascorbate peroxidase 1 [*Arabidopsis* *thaliana*]), (**c**) guaiacol peroxidase (peroxidase PPOD1-like precursor [*Hydra* *vulgaris*]) and (**d**) catalase (catalase 3 [*Arabidopsis* *thaliana*]).

. The gene (Zm00001d010039) showed significant drop-offs during periods devoid of adequate water supply. Catalase enzyme is controlled by a particular gene, catalase 3 [*Arabidopsis* *thaliana*]. Catalase 3 [*Arabidopsis* *thaliana*] expression was studied under drought stress^29^. The findings illustrated that each gene related to the actions of the precursor enzyme typically showcased an expression decline when facing drought conditions; intriguingly, no basic gene demonstrated substantial declines during times lacking sufficient water availability (Fig. 2).

**Figure 3:**
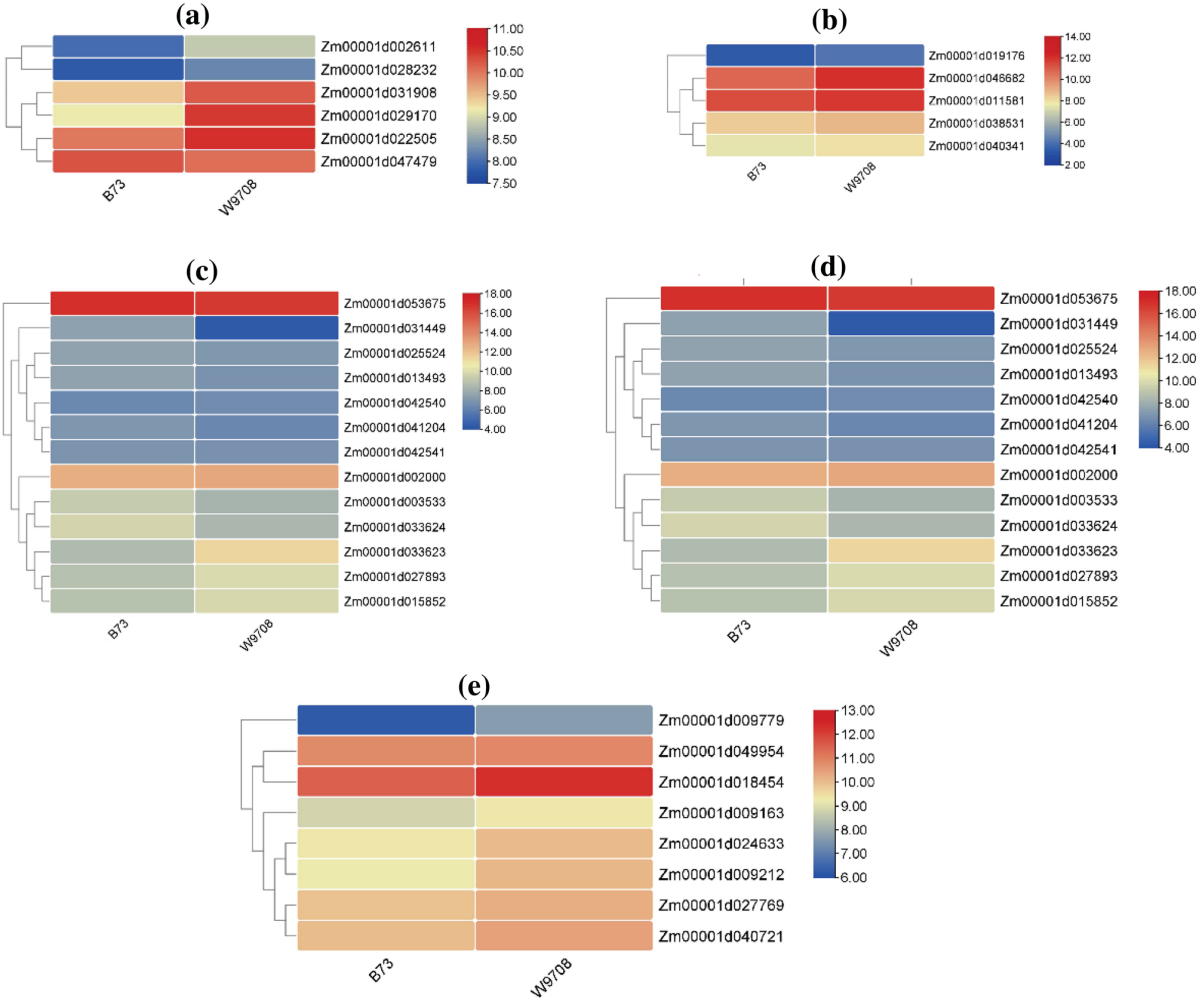
Gene expression level of (**a**) lipid peroxidation (peroxiredoxin, type II [*Volvox* *carteri* f. nagariensis]), (**b**) Superoxide dismutase (copper/zinc superoxide dismutase 1 [*Arabidopsis* *thaliana*]), (**c**) lipoxygenase (lipoxygenase 1 [*Arabidopsis* *thaliana*]), (**d**) glutathione-*S*-transferase (glutathione *S*-transferase 6 [*Arabidopsis* *thaliana*]) and (**e**) glutathione reductase (glutathione reductase [*Arabidopsis* *thaliana*]).

Lipid peroxidation/malondialdehyde(MDA) enzyme regulated by gene peroxiredoxin, type II [*Volvox* *carteri* f. nagariensis]^30^. The regulation mechanism was studied in dry conditions. Findings indicated that all genes tied to the enzyme's activities mentioned above generally experienced reduced expression under drought conditions. Superoxide dismutase enzyme activity was also studied in dry conditions. The transcriptome of the key gene copper/zinc superoxide dismutase 1 [*Arabidopsis* *thaliana*] was done. In particular, one gene (Zm00001d019176) exhibited significant decreases during times lacking sufficient hydration^31^. Lipoxygenase enzyme was controlled with gene lipoxygenase 1 [Arabidopsis thaliana], and transcriptome analysis was performed in drought stress. Each gene associated with the activities of the lipoxygenase enzyme generally exhibited lessened expression in drier conditions^32,33^. Specifically, five genes (Zm00001d003533, Zm00001d031449, Zm00001d041204, Zm00001d053675, and ZM00225) saw considerable reductions during times lacking sufficient water supply. Glutathione-S-transferase is managed by gene glutathione S-transferase 6 [*Arabidopsis* *thaliana*]^34^. Five genes (Zm00001d002000, Zm00001d031449, Zm00001d041204, Zm00001d053675, and Zm00001d015852) demonstrated significant decreases during drought stress. Glutathione Reductase enzyme controlled with glutathione reductase [*Arabidopsis* *thaliana*]^35^. Only one gene (Zm00001d009212) exhibited significant reductions during intervals lacking sufficient water supply (Fig. 3).

## Discussion

Soil salinity can impact every plant growth and development stage, including seedling establishment, germination, vegetative growth, and reproductive phases. Among these stages, seed germination is particularly vital and is influenced by genetic factors and environmental cues^36^. The percentage of seeds that successfully germinate, known as Seed Germination Percentage (SGP), is significantly influenced by varying levels of sodium chloride (NaCl) in the soil^37^, leading to a decrease in the growth of maize plants. While no significant reduction was observed at 50 mM NaCl compared to the control group, a noticeable decline was recorded at higher concentrations of 100 and 150 mM NaCl, respectively.

However, it's important to note that the most significant decrease in seed germination percentage (SGP) was observed at 150 mM NaCl concentration. This highlights that 150 mM NaCl harms seed germination, although maize plants can manage seed survival even under this high salt concentration. Seed germination relies on water and occurs in three distinct phases. Insufficient water availability profoundly impacts the metabolic processes related to germination, leading to a failure in seed germination^38^. Salt-induced stress interferes with the proper development of the embryonic axis, resulting in a reduced osmotic potential in the growth medium and restricted water availability within the seeds^39^. Nonetheless, the successful emergence of the initial root in response to osmotic pressure, triggered by reduced water availability due to high salt concentrations (150 mM NaCl) in maize seeds, requires a decrease in the suppression of germination potential (SGP). To counteract the adverse effects of salt on seed germination, various factors have been proposed to play a role in promoting successful germination, including increased seed reserves, elevated levels of gibberellic acid within the seed, efficient carbohydrate metabolism, enhanced antioxidant defense mechanisms, activation of MAPK signaling^40^. However, due to limited available data in the context of maize, it remains challenging to dissect the contributions of each of these elements individually.

The subsequent critical stage in achieving successful seed germination is the establishment of the seedling, a phase that profoundly influences overall plant productivity^41^. In saline environments, the presence of ROS within plant cells is instigated by salt's osmotic and ionic effects^42^. Numerous studies have demonstrated the accumulation of hydrogen peroxide (H_2_O_2_) in the leaves of plant species sensitive to salt stress^43^. Interestingly, in salt-tolerant pea cultivars, H_2_O_2_ levels either decreased or exhibited restrained growth^44^.

The activity of numerous enzymes is influenced by H_2_O_2_, which directly or indirectly oxidizes pools of antioxidants like ASA and GSH, resulting in the buildup of oxidized redox compounds within cells for various cellular processes^45^. SODs exist in various isoforms (Cu–Zn-SOD/Fe-SOD/Mn-SOD), responding to the presence of H_2_O_2_, and are located within cellular organelles such as the cytoplasm, mitochondria and chloroplasts^46^. High salinity conditions stimulate the plasma membrane, generating highly toxic superoxide radicals (O~~) with a short lifespan, catalyzed by NADPH oxidase^47^. The chloroplast and mitochondrial electron transport chains also contribute to O~~ production under saline conditions^48^.

In saline environments, limited water availability leads to stomatal closure and reduced carbon dioxide assimilation. This prompts the transfer of electrons to molecular oxygen to generate O~~, reducing the availability of the electron acceptor NADP+^49^. SODs play a crucial role in dismutating extremely toxic O~~ into the less harmful hydrogen peroxide (H_2_O_2_)^50^. Interestingly, the increase in specific SOD activity observed in maize leaves is attributed to heightened activities of other enzymes like CAT, GPOD, and APOD within these leaves. A corresponding increase in H_2_O_2_ levels does not accompany this rise in SOD-specific activity.

The activity of SOD increased consistently, by 1.5 times, in the leaves exposed to 100 mM NaCl, thereby protecting against harmful effects. This heightened SOD activity is an adaptive response that enhances salt tolerance^50^. In sorghum seedlings subjected to saline conditions, elevated gene expression was observed for four SOD genes (SOD-Fe1, Sod-Cu–Zn-4A, SOD-Cu–Zn-2, and SOD-Cu-Mn), underscoring the critical role of SODs in managing ROS^51^. Salinity-induced ROS, including H_2_O_2_, contribute to lipid peroxidation of membranes^52,53^.

Furthermore, at 80 mM NaCl, Lipoxygenase activity notably decreased, as contributing to the lower MDA levels. However, at 100 mM NaCl, LOX activity increased, yet the MDA levels remained low compared to control conditions. The reduced MDA levels at 100 Mm NaCl might be attributed to higher levels of Glutathione-S-Transferase (GST) (34 nmol min^−1^ mg^−1^ protein) compared to controls (26 nmol min^−1^ mg^−1^ protein). GST can rapidly scavenge lipid peroxides through its peroxidative activity^54^. Through the combined action of peroxidative enzymes, namely CAT, GPOD and APOD the levels of H_2_O_2_ are reduced in maize, maintaining a steady state. The activity of CAT, a pivotal enzyme in H_2_O_2_ detoxification, increased as compared to controls at 80 mM NaCl, but subsequently decreased at 100 mM NaCl. CAT efficiently scavenges excess H_2_O_2_ due to its affinity for the molecule^55^. At 80 mM NaCl, CAT exhibited robust H_2_O_2_ clearance. Its diminished activity (0.08 nmole min^−1^ mg^−1^ protein) at 100 mM NaCl is compensated by the heightened activities of APOD (0.30 nmol min^−1^ mg^−1^ protein) and GPOD (0.19 nmol min^−1^ mg^−1^ protein) at the same concentration of NaCl. CAT's protective role in maize leaves appears to be effective only up to 80 mM NaCl. It has been demonstrated that synthesizing a specific form of CAT becomes limited at higher salt concentrations^56^. Notably, transgenic plants expressing both the GhSOD1 and GhCAT1 genes in cotton exhibited enhanced tolerance to salt stress (200 mM NaCl)^57^. This suggests that coordinated action between SOD and CAT genes could confer increased resistance to high salt pressure.

GPODs play a significant role in various plant functions, including defense against pathogens, reinforcement of cell walls, auxin metabolism, lignin synthesis, and tolerance to abiotic stresses^58^. Importantly, GPODs efficiently detoxify H_2_O_2_ using a non-specific electron donor (2RH + H_2_O_2_ → 2R⋅ + 2H_2_O)^59^. In the context of salt-stressed leaves, as H_2_O_2_ and MDA levels decrease, GPOD levels consistently increase with rising NaCl concentrations. The elevation in GPOD activity was lower at 50 mM, 80 mM, as compared to 100 mM while higher as compared to control leaves. Notably, GPOD activity remained constant across all tested NaCl concentrations in the internode and can be considered a hallmark of stress tolerance. In sorghum seedlings subjected to salt stress, an increased expression of three GPOD genes (POD-2E-1, POD-2F, POD-2C) has been observed, emphasizing the role of GPODs in salt response^60^.

Moreover, in wheat, higher levels of GPOD, SOD and CAT, accompanied by a rapid decrease in ROS and MDA, were achieved through the overexpression of the TaPRX-2A gene, leading to enhanced salt tolerance^61^. Based on recent findings in the internodal husk (IH) and other reports, it is evident that GPOD is crucial for maintaining lower levels of H_2_O_2_, thereby contributing to salt tolerance in saline conditions. Due to its strong affinity for H_2_O_2_ and APOD are a more efficient scavenger of H_2_O_2_ compared to CAT, making it a crucial regulator of H_2_O_2_ levels in plant cells^62^. Unlike the decline in APOD activity observed at 80 mM NaCl, APOD activity increased in leaves exposed to 80 mM NaCl and then only slightly decreased at 100 mM NaCl, remaining elevated as compared to controls. The higher levels of APOD corresponded to the decreasing levels of H_2_O_2_ and MDA in maize leaves. APOD collaborates with Glutathione Reductase (GR) to detoxify H_2_O_2_ into water, indirectly relying on a pool of Ascorbic Acid^63^.

The impact of the APOD-GR pathway on H_2_O_2_ regulation can be attributed to its presence in various cellular compartments involved in ROS production, including the cytoplasm, chloroplasts, mitochondria, and peroxisomes^64^. The reduction in APOD activity and slight increase in GR activity at 100 mM NaCl might result from the harmful effects of Na^+^ and Cl^-^ ions, or the limited availability of NADPH due to impaired photosynthetic machinery under salt stress^65^. GR utilizes NADPH to convert oxidized glutathione (GSSG) to reduced glutathione (GSH), catalyzing the final and rate-limiting step of the APOD-GR pathway^66^. Even under 100 mM NaCl conditions, these subtle changes in APOD and GR activities, along with maintained low H_2_O_2_ levels, were observed in the leaves of maize, reflecting the collaborative nature of different antioxidant enzymes in ROS regulation within the internodal husk fibre.

In the salt-tolerant maize cultivar (BR5033), higher levels of APOD and GR activities were observed compared to the salt-sensitive cultivar (BR5011)^67^. In wheat, GST gene expression was reduced at 100 mM NaCl and moderately upregulated at 200 mM NaCl, whereas it was significantly increased at the higher salt concentration of 300 mM NaCl^68^.

The observed variations in responses to salt stress among different plant species might be attributed to differences in Glutathione S-Transferase (GST) gene expression. GST genes have been found to play a critical role in safeguarding plants against Reactive Oxygen Species (ROS)^69^. Through their peroxidative activity, GSTs are capable of scavenging various ROS, including superoxide radicals (O~~), hydrogen peroxide (H_2_O_2_), hydroxyl radicals (OH⋅), and lipid peroxides^70^. Recent research has suggested a dual role for the GSTU7 gene, which contributes to both restraining plant growth and detoxifying ROS through its glutathione peroxidase activity, thereby enhancing resistance to oxidative stress in *Arabidopsis* *thaliana*^70^.

Indeed, the variations in responses to salt stress across different plant species can often be attributed to the distinct expression patterns of glutathione *S*-transferase (GST) genes. GSTs are critical in the plant's defense against reactive oxygen species (ROS)^71^. These enzymes, through their peroxidative activity, are capable of effectively scavenging a wide range of ROS, including superoxide radicals (O~~), hydrogen peroxide (H_2_O_2_), hydroxyl radicals (OH⋅), and lipid peroxides^69^. The higher genetic advance indicated that the gene expression in maize genotype may be fixed in the next generation to improve abiotic stress tolerance in maize.

The research findings during gene expression level suggest that drought stress has a consistent suppressive effect on the expression of genes associated with various enzymes involved in oxidative stress responses in maize strains. Specifically, hydrogen peroxide (H_2_O_2_) regulation genes, such as peroxidase 4-like [*Momordica* *charantia*]^25^, were generally down-regulated under dryer conditions, indicating a reduced ability to counteract oxidative stress. Similarly, genes linked to the expression of enzymes like ascorbate peroxidase and guaiacol peroxidase^26,27^ exhibited significant down-regulation during insufficient water availability, implying a diminished capacity to scavenge ROS. The study also revealed that the expression of genes controlling the Catalase enzyme^29^ remained relatively stable under drought stress, suggesting a differential response among different antioxidant systems in maize strains.

Genes responsible for regulating enzymes involved in lipid peroxidation^30^, Superoxide dismutase^31^, and Lipoxygenase^32^, demonstrated a reduced expression pattern in drier conditions, highlighting the maize plants' challenge in managing oxidative stress during drought.

Lastly, genes controlling glutathione-*S*-transferase^34^ and glutathione reductase^35^ enzymes displayed significant decreases in expression during drought stress, indicating potential disruptions in the cellular antioxidant defense mechanisms^72–74^. These findings underscore the maize strains' vulnerability to oxidative stress under drought conditions and shed light on the genetic regulation of key antioxidant enzymes in response to water scarcity.

## Conclusion

Plant responses to salt stress involve intricate antioxidant enzyme systems, such as SODs, CAT, GPOD, and APOD, which collaborate to regulate ROS levels. GST genes play a vital role in ROS detoxification. Gene expression and enzymatic activity variations contribute to diverse salt stress responses across plant species, showcasing complex adaptation strategies. The study also highlights the vulnerability of maize strains to oxidative stress under drought conditions. The down-regulation of genes controlling antioxidant enzymes suggests that these plants face challenges managing reactive oxygen species during water scarcity. Understanding the genetic regulation of antioxidant systems can be crucial for developing drought-resistant maize varieties and improving crop resilience in changing climates. While higher genetic advance revealed that the gene expression may be fixed for next generation, hence the selection of maize genotype as abiotic tolerance may be helpful to develop stress tolerant maize genotypes and hybrids.

**Table:** 

Sr	Enzyme	Genes families (NCBI)	NCBI reference sequence
1	Hydrogen peroxide (H_2_O_2_)	Peroxidase 4-like [*Momordica* *charantia*]	XP_022149597.1
2	Lipid peroxidation/malondialdehyde (MDA)	Peroxiredoxin, type II [*Volvox* *carteri* f. *nagariensis*]	XP_002953605.1
3	Superoxide dismutase	Copper/zinc superoxide dismutase 1 [*Arabidopsis* *thaliana*]	NP_172360.1
4	Lipoxygenase	Lipoxygenase 1 [*Arabidopsis* *thaliana*]	NP_175900.1
5	Glutathion-*S*-transferase	Glutathione *S*-transferase 6 [*Arabidopsis* *thaliana*]	NP_001184893.1
6	Glutathion reductase	Glutathione reductase [*Arabidopsis* *thaliana*]	AEE79262.1
7	Ascorbate peroxidase	Ascorbate peroxidase 1 [*Arabidopsis* *thaliana*]	NP_172267.1
8	Guaiacol peroxidase	Peroxidase PPOD1-like precursor [*Hydra* *vulgaris*]	NP_001267756.1
9	Catalase	Catalase 3 [*Arabidopsis* *thaliana*]	NP_564120.1

### Supplementary Information

Supplementary Information 1.

Supplementary Information 2.

Supplementary Information 3.

## Data Availability

The produced, collected, or generated during the study has been given in the manuscript file and supplementary material files. I affirm that all necessary data and permissions have been provided for this study. Any interested researchers can access the required data to support the findings and conclusions of this article. For publicly archived datasets, hyperlinks are provided in this manuscript in appropriate places for convenience.
