# Supplementary material for: Antioxidant production promotes defense mechanism and different gene expression level in Zea mays under abiotic stress
Source: Sci Rep. 2024 Mar 26;14:7114. doi: 10.1038/s41598-024-57939-6 (PMC10965962; doi:10.1038/s41598-024-57939-6)
Supplement: Supplementary file 1 — Supplementary Information 1. [file 41598_2024_57939_MOESM1_ESM.docx]

**File S1**

**Supplementary Material ANOVA tables under salt stress conditions**

**Randomized Complete Block AOV Table for Catalase**

**Source DF SS MS F P**

Replicati 2 0.00233 0.00117

Treatment 5 0.01380 0.00276 8.0E+31 0.0000

Error 8 2.764E-34 3.456E-35

Total 15

**Randomized Complete Block AOV Table for Gultathione-S- transferase**

**Source DF SS MS F P**

Replicati 2 0.00126 6.281E-04

Treatment 5 6.000E-04 1.200E-04 9.0E+30 0.0000

Error 8 1.069E-34 1.337E-35

Total 15

**Randomized Complete Block AOV Table for Glutathione reductase**

**Source DF SS MS F P**

Replicati 2 0.00103 0.00051

Treatment 5 0.00670 0.00134 15.57 0.0006

Error 8 0.00069 0.00009

Total 15

**Randomized Complete Block AOV Table for Guaiacol peroxidase**

**Source DF SS MS F P**

Replicati 2 0.00402 0.00201

Treatment 5 0.00727 0.00145 24.48 0.0001

Error 8 0.00048 0.00006

Total 15

**Randomized Complete Block AOV Table for Ascorbate peroxidase**

**Source DF SS MS F P**

Replicati 2 0.00643 0.00322

Treatment 5 0.00779 0.00156 34.62 0.0000

Error 8 0.00036 0.00005

Total 15

**Randomized Complete Block AOV Table for H_2_O_2_**

**Source DF SS MS F P**

Replicati 2 0.00400 0.00200

Treatment 5 47162.3 9432.47 3.2E+31 0.0000

Error 8 2.344E-27 2.930E-28

Total 15

**Randomized Complete Block AOV Table for Leaf area**

**Source DF SS MS F P**

Replicati 2 0.00400 0.00200

Treatment 5 2.78823 0.55765 2.9E+30 0.0000

Error 8 1.529E-30 1.911E-31

Total 15

**Randomized Complete Block AOV Table for MDA**

**Source DF SS MS F P**

Replicati 2 0.00400 0.00200

Treatment 5 331.389 66.2778 1.9E+32 0.0000

Error 8 2.748E-30 3.434E-31

Total 15

**Randomized Complete Block AOV Table for Lipidoxygenase**

**Source DF SS MS F P**

Replicati 2 0.00400 0.00200

Treatment 5 4744.37 948.874 2.3E+31 0.0000

Error 8 3.248E-28 4.060E-29

Total 15

**Randomized Complete Block AOV Table for No of roots**

**Source DF SS MS F P**

Replicati 2 0.00400 0.00200

Treatment 5 38.5044 7.70089 9.3E+30 0.0000

Error 8 6.618E-30 8.272E-31

Total 15

**Randomized Complete Block AOV Table for Root length**

**Source DF SS MS F P**

Replicati 2 0.00400 0.00200

Treatment 5 68.6506 13.7301 1.5E+31 0.0000

Error 8 7.567E-30 9.459E-31

Total 15

**Randomized Complete Block AOV Table for Root weight**

**Source DF SS MS F P**

Replicati 2 0.00400 0.00200

Treatment 5 1.356E-04 2.712E-05 3.4E+29 0.0000

Error 8 6.459E-34 8.074E-35

Total 15

**Randomized Complete Block AOV Table for Root to shoot length ratio**

**Source DF SS MS F P**

Replicati 2 0.00400 0.00200

Treatment 5 0.06319 0.01264 2.0E+31 0.0000

Error 8 5.066E-33 6.333E-34

Total 15

**Randomized Complete Block AOV Table for Seed germination %age**

**Source DF SS MS F P**

Replicati 2 0.00400 0.00200

Treatment 5 4583.81 916.763 7.6E+31 0.0000

Error 8 9.684E-29 1.210E-29

Total 15

**Randomized Complete Block AOV Table for Shoot length**

**Source DF SS MS F P**

Replicati 2 0.00400 0.00200

Treatment 5 3.90433 0.78087 1.3E+30 0.0000

Error 8 4.743E-30 5.928E-31

Total 15

**Randomized Complete Block AOV Table for Superoxide dismutase**

**Source DF SS MS F P**

Replicati 2 0.00400 0.00200

Treatment 5 53.7058 10.7412 6.6E+31 0.0000

Error 8 1.295E-30 1.619E-31

Total 15

**Randomized Complete Block AOV Table for leaf length**

**Source DF SS MS F P**

Replicati 2 0.00400 0.00200

Treatment 5 0.30000 0.06000 8.9E+29 0.0000

Error 8 5.418E-31 6.773E-32

Total 15

**Randomized Complete Block AOV Table for leaf weight**

**Source DF SS MS F P**

Replicati 2 0.00400 0.00200

Treatment 5 0.33411 0.06682 2.0E+30 0.0000

Error 8 2.694E-31 3.367E-32

Total 15

**Randomized Complete Block AOV Table for shoot weight**

**Source DF SS MS F P**

Replicati 2 0.00400 0.00200

Treatment 5 5.792E-04 1.158E-04 3.2E+29 0.0000

Error 8 2.920E-33 3.650E-34

Total 15

**Genetic components for various traits of maize under salt and drought stress conditions**

| **Traits** | **M.S** | **G.M** | **GV** | **GCV %** | **PV** | **PCV %** | **EV** | **ECV %** | **h2bs%** | **GA%** |
| --- | --- | --- | --- | --- | --- | --- | --- | --- | --- | --- |
| **Root length** | 13.7301* | 12.355 | 4.577 | 60.863 | 4.577 | 60.864 | 0.0001 | 0.284 | 99.998 | 80.629 |
| **Root weight** | 0.0003* | 0.102 | 0.000 | 1.808 | 0.000 | 4.783 | 0.0002 | 4.428 | 14.286 | 2.395 |
| **Root-to-shoot length ratio** | 0.0127* | 0.394 | 0.004 | 10.361 | 0.004 | 10.374 | 0.0000 | 0.504 | 99.764 | 13.727 |
| **Seed germination %age** | 916.763* | 55.656 | 305.588 | 234.321 | 305.588 | 234.321 | 0.0001 | 0.134 | 100.000 | 310.421 |
| **shoot length** | 0.781* | 7.863 | 0.260 | 18.193 | 0.260 | 18.200 | 0.0002 | 0.504 | 99.923 | 24.102 |
| **SOD** | 10.742* | 4.098 | 3.581 | 93.474 | 3.581 | 93.477 | 0.0002 | 0.699 | 99.994 | 123.831 |
| **Leaf length** | 0.060* | 2.719 | 0.019 | 8.403 | 0.022 | 8.913 | 0.0024 | 2.971 | 88.889 | 11.132 |
| **Leaf width** | 0.0668* | 2.146 | 0.022 | 10.170 | 0.022 | 10.218 | 0.0002 | 0.989 | 99.063 | 13.473 |
| **Shoot weight** | 0.0004* | 0.207 | 0.000 | 2.198 | 0.000 | 3.108 | 0.0001 | 2.198 | 50.000 | 2.912 |
| **catalase** | 0.0028* | 0.098 | 0.001 | 9.512 | 0.001 | 10.034 | 0.0001 | 3.194 | 89.865 | 12.601 |
| **Gultathione-S- transferase** | 0.0004* | 0.059 | 0.000 | 4.694 | 0.000 | 4.871 | 0.0001 | 1.302 | 92.857 | 6.218 |
| **Glutathione reductase** | 0.0013* | 0.084 | 0.000 | 7.015 | 0.001 | 7.817 | 0.0001 | 3.450 | 80.519 | 9.293 |
| **Guaiacol peroxidase** | 0.0015* | 0.075 | 0.000 | 7.746 | 0.001 | 8.563 | 0.0001 | 3.651 | 81.818 | 10.262 |
| **Ascorbate peroxidase** | 0.0016* | 0.076 | 0.000 | 8.002 | 0.001 | 8.786 | 0.0001 | 3.627 | 82.955 | 10.601 |
| **H2O2** | 9432.470* | 150.847 | 3144.157 | 456.545 | 3144.157 | 456.545 | 0.0002 | 0.115 | 100.000 | 604.815 |
| **Leaf area** | 0.558* | 4.233 | 0.186 | 20.962 | 0.186 | 20.962 | 0.0001 | 0.154 | 99.995 | 27.769 |
| **MDA** | 66.278* | 9.315 | 22.093 | 154.004 | 22.093 | 154.004 | 0.0001 | 0.104 | 100.000 | 204.019 |
| **Lipoxygenase** | 948.847* | 43.650 | 316.282 | 2.692 | 316.282 | 0.407 | 0.0001 | 0.151 | 100.000 | 356.602 |
| **No of roots** | 7.701* | 6.566 | 2.567 | 0.625 | 2.567 | 0.244 | 0.0000 | 0.123 | 100.000 | 82.833 |

*=Significant at 5% probability level, Mean Sum of Squares (M.S), Grand mean (G.M), Genotypic variance (GV), Genotypic coefficient of variance (GCV %), Phenotypic variance (PV), Phenotypic coefficient of variance (PCV %), Environmental Variance (EV), Environmental coefficient of variance (ECV %), Broad sense heritability (h2bs %), Genetic advance
