# Supplementary material for: Antioxidant production promotes defense mechanism and different gene expression level in Zea mays under abiotic stress
Source: Sci Rep. 2024 Mar 26;14:7114. doi: 10.1038/s41598-024-57939-6 (PMC10965962; doi:10.1038/s41598-024-57939-6)
Supplement: Supplementary file 2 — Supplementary Information 2. [file 41598_2024_57939_MOESM2_ESM.docx]

**File S2**

**Supplementary Material ANOVA tables under Drought stress conditions**

**Randomized Complete Block AOV Table for Catalase**

**Source DF SS MS F P**

Replicati 2 1.867E-05 9.333E-06

Treatment 4 0.01015 0.00254 5.0E+31 0.0000

Error 6 3.046E-34 5.076E-35

Total 12

**Randomized Complete Block AOV Table for Gultathione-S- transferase**

**Source DF SS MS F P**

Replicati 2 0.00979 0.00490

Treatment 4 0.03043 0.00761 3.16 0.1013

Error 6 0.01445 0.00241

Total 12

**Randomized Complete Block AOV Table for Glutathione reductase**

**Source DF SS MS F P**

Replicati 2 0.00615 0.00307

Treatment 4 0.00482 0.00121 2.11 0.1969

Error 6 0.00342 0.00057

Total 12

**Randomized Complete Block AOV Table for Guaiacol peroxidase**

**Source DF SS MS F P**

Replicati 2 0.00229 0.00115

Treatment 4 0.12082 0.03020 7.0E+31 0.0000

Error 6 2.589E-33 4.314E-34

Total 12

**Randomized Complete Block AOV Table for Ascorbate peroxidase**

**Source DF SS MS F P**

Replicati 2 0.01537 0.00769

Treatment 4 0.07403 0.01851 7.68 0.0153

Error 6 0.01445 0.00241

Total 12

**Randomized Complete Block AOV Table for H_2_O_2_**

**Source DF SS MS F P**

Replicati 2 0.01482 0.00741

Treatment 4 94242.5 23560.6 9782956 0.0000

Error 6 0.01445 0.00241

Total 12

**Randomized Complete Block AOV Table for Leaf area**

**Source DF SS MS F P**

Replicati 2 0.01482 0.00741

Treatment 4 6.03036 1.50759 625.99 0.0000

Error 6 0.01445 0.00241

Total 12

**Randomized Complete Block AOV Table for MDA**

**Source DF SS MS F P**

Replicati 2 0.015 0.0074

Treatment 4 354.800 88.7000 36830.4 0.0000

Error 6 0.014 0.0024

Total 12

**Randomized Complete Block AOV Table for Lipidoxygenase**

**Source DF SS MS F P**

Replicati 2 0.01 0.007

Treatment 4 2277.71 569.428 236441 0.0000

Error 6 0.01 0.002

Total 12

**Randomized Complete Block AOV Table for No of roots**

**Source DF SS MS F P**

Replicati 2 0.0148 0.00741

Treatment 4 17.2708 4.31771 1792.82 0.0000

Error 6 0.0144 0.00241

Total 12

**Randomized Complete Block AOV Table for Root length**

**Source DF SS MS F P**

Replicati 2 0.0148 0.0074

Treatment 4 69.1507 17.2877 7178.28 0.0000

Error 6 0.0145 0.0024

Total 12

**Randomized Complete Block AOV Table for Root weight**

**Source DF SS MS F P**

Replicati 2 0.01482 0.00741

Treatment 4 0.04783 0.01196 4.97 0.0413

Error 6 0.01445 0.00241

Total 12

**Randomized Complete Block AOV Table for Root to shoot length ratio**

**Source DF SS MS F P**

Replicati 2 0.01482 0.00741

Treatment 4 0.70067 0.17517 72.73 0.0000

Error 6 0.01445 0.00241

Total 12

**Randomized Complete Block AOV Table for Seed germination %age**

**Source DF SS MS F P**

Replicati 2 0.01 0.007

Treatment 4 2213.69 553.422 229795 0.0000

Error 6 0.01 0.002

Total 12

**Randomized Complete Block AOV Table for Shoot length**

**Source DF SS MS F P**

Replicati 2 0.015 0.0074

Treatment 4 101.394 25.3484 10525.3 0.0000

Error 6 0.014 0.0024

Total 12

**Randomized Complete Block AOV Table for Superoxide dismutase**

**Source DF SS MS F P**

Replicati 2 0.0148 0.00741

Treatment 4 29.8337 7.45843 3096.93 0.0000

Error 6 0.0145 0.00241

Total 12

**Randomized Complete Block AOV Table for leaf length**

**Source DF SS MS F P**

Replicati 2 0.01482 0.00741

Treatment 4 0.42014 0.10504 43.61 0.0001

Error 6 0.01445 0.00241

Total 12

**Randomized Complete Block AOV Table for leaf weight**

**Source DF SS MS F P**

Replicati 2 0.01482 0.00741

Treatment 4 0.43972 0.10993 45.65 0.0001

Error 6 0.01445 0.00241

Total 12

**Randomized Complete Block AOV Table for shoot weight**

**Source DF SS MS F P**

Replicati 2 0.01482 0.00741

Treatment 4 0.06292 0.01573 6.53 0.0224

Error 6 0.01445 0.00241

Total 12

**Genetic components for various traits of maize under drought stress conditions**

| **Traits** | **M.S** | **G.M** | **GV** | **GCV %** | **PV** | **PCV %** | **EV** | **ECV %** | **h^2^bs%** | **GA%** |
| --- | --- | --- | --- | --- | --- | --- | --- | --- | --- | --- |
| **Root length** | 17.288* | 12.593 | 5.762 | 67.642 | 5.764 | 67.656 | 0.002 | 1.381 | 99.958 | 89.610 |
| **Root weight** | 0.012* | 0.319 | 0.003 | 9.911 | 0.006 | 13.289 | 0.003 | 8.853 | 55.621 | 13.129 |
| **Root-to-shoot length ratio** | 0.175* | 1.268 | 0.058 | 21.301 | 0.060 | 21.777 | 0.003 | 4.528 | 95.676 | 28.219 |
| **Seed germination %age** | 553.422* | 69.943 | 184.473 | 162.403 | 184.475 | 162.404 | 0.002 | 0.535 | 99.999 | 215.146 |
| **shoot length** | 25.348* | 6.261 | 8.448 | 116.162 | 8.451 | 116.183 | 0.003 | 2.189 | 99.965 | 153.887 |
| **SOD** | 7.458* | 4.243 | 2.485 | 76.532 | 2.488 | 76.570 | 0.003 | 2.427 | 99.900 | 101.386 |
| **Leaf length** | 0.105* | 2.759 | 0.034 | 11.139 | 0.037 | 11.523 | 0.002 | 2.949 | 93.449 | 14.757 |
| **Leaf width** | 0.110* | 2.439 | 0.036 | 12.132 | 0.038 | 12.498 | 0.002 | 3.003 | 94.226 | 16.072 |
| **Shoot weight** | 0.016* | 0.376 | 0.004 | 10.899 | 0.007 | 13.415 | 0.002 | 7.821 | 66.010 | 14.439 |
| **catalase** | 0.003* | 0.094 | 0.001 | 9.312 | 0.001 | 9.868 | 0.000 | 3.265 | 89.051 | 12.336 |
| **Gultathione-S- transferase** | 0.008* | 0.078 | 0.002 | 14.879 | 0.004 | 23.004 | 0.002 | 17.544 | 41.834 | 19.711 |
| **Glutathione reductase** | 0.001* | 0.053 | 0.000 | 6.194 | 0.001 | 12.311 | 0.001 | 10.640 | 25.311 | 8.205 |
| **Guaiacol peroxidase** | 0.030* | 0.048 | 0.010 | 45.491 | 0.010 | 46.398 | 0.000 | 9.129 | 96.129 | 60.265 |
| **Ascorbate peroxidase** | 0.019* | 0.067 | 0.005 | 28.293 | 0.008 | 34.062 | 0.002 | 18.966 | 68.997 | 37.482 |
| **H_2_O_2_** | 23560.600* | 132.230 | 7853.532 | 770.668 | 7853.536 | 770.669 | 0.003 | 0.509 | 99.970 | 1020.954 |
| **Leaf area** | 1.508* | 4.868 | 0.502 | 32.107 | 0.504 | 32.184 | 0.002 | 2.225 | 99.522 | 42.535 |
| **MDA** | 88.700* | 8.586 | 29.553 | 185.525 | 29.595 | 185.657 | 0.042 | 6.994 | 99.858 | 245.777 |
| **Lipoxygenase** | 569.428* | 28.745 | 189.809 | 2.570 | 189.811 | 0.479 | 0.002 | 0.834 | 99.999 | 340.420 |
| **No of roots** | 4.318* | 9.993 | 1.438 | 0.379 | 1.441 | 0.120 | 0.002 | 1.550 | 99.833 | 50.262 |

*=Significant at 5% probability level, Mean Sum of Squares (M.S), Grand mean (G.M), Genotypic variance (GV), Genotypic coefficient of variance (GCV %), Phenotypic variance (PV), Phenotypic coefficient of variance (PCV %), Environmental Variance (EV), Environmental coefficient of variance (ECV %), Broad sense heritability (h^2^bs %), Genetic advance (GA)
