# Supplementary material for: Antioxidant production promotes defense mechanism and different gene expression level in Zea mays under abiotic stress
Source: Sci Rep. 2024 Mar 26;14:7114. doi: 10.1038/s41598-024-57939-6 (PMC10965962; doi:10.1038/s41598-024-57939-6)
Supplement: Supplementary file 3 — Supplementary Information 3. [file 41598_2024_57939_MOESM3_ESM.docx]

**File S3**

**Supplementary Material combined ANOVA under drought and salt stress conditions**

**Randomized Complete Block ANOVA Table for Root length**

**Source DF SS MS F P**

Replication 2 0.017 0.0084

Treatment 9 226.185 25.1317 26088.3 0.0000

Error 18 0.017 0.0010

Total 29 226.219

Grand Mean 12.182 CV 0.25

**Randomized Complete Block ANOVA Table for Root weight**

**Source DF SS MS F P**

Replication 2 0.01673 0.00836

Treatment 9 0.41124 0.04569 47.43 0.0000

Error 18 0.01734 0.00096

Total 29 0.44530

Grand Mean 0.1976 CV 15.71

**Randomized Complete Block ANOVA Table for Root-to-shoot length ratio**

**Source DF SS MS F P**

Replication 2 0.01673 0.00836

Treatment 9 6.38499 0.70944 736.45 0.0000

Error 18 0.01734 0.00096

Total 29 6.41906

Grand Mean 0.7798 CV 3.98

**Randomized Complete Block ANOVA Table for Seed germination %age**

**Source DF SS MS F P**

Replication 2 0.02 0.00836

Treatment 9 9862.08 1095.79 1137494 0.0000

Error 18 0.02 9.633E-04

Total 29 9862.11

Grand Mean 63.270 CV 0.05

**Randomized Complete Block ANOVA Table for Shoot length**

**Source DF SS MS F P**

Replication 2 0.017 0.0084

Treatment 9 123.892 13.7658 14289.8 0.0000

Error 18 0.017 0.0010

Total 29 123.926

Grand Mean 7.1517 CV 0.43

**Randomized Complete Block ANOVA Table for Superoxid dismutase**

**Source DF SS MS F P**

Replication 2 0.0167 0.0084

Treatment 9 94.2263 10.4696 10868.1 0.0000

Error 18 0.0173 0.0010

Total 29 94.2604

Grand Mean 4.0671 CV 0.76

**Randomized Complete Block ANOVA Table for leaf length**

**Source DF SS MS F P**

Replication 2 0.01673 0.00836

Treatment 9 0.73347 0.08150 84.60 0.0000

Error 18 0.01734 0.00096

Total 29 0.76754

Grand Mean 2.7377 CV 1.13

**Randomized Complete Block ANOVA Table for leaf width**

**Source DF SS MS F P**

Replication 2 0.01673 0.00836

Treatment 9 1.40960 0.15662 162.58 0.0000

Error 18 0.01734 0.00096

Total 29 1.44367

Grand Mean 2.2767 CV 1.36

**Randomized Complete Block ANOVA Table for shoot weight**

**Source DF SS MS F P**

Replication 2 0.01673 0.00836

Treatment 9 0.28084 0.03120 32.39 0.0000

Error 18 0.01734 0.00096

Total 29 0.31490

Grand Mean 0.2818 CV 11.02

**Randomized Complete Block ANOVA Table for Catalase**

**Source DF SS MS F P**

Replication 2 0.03623 0.01811

Treatment 9 0.02316 0.00268 6.42 0.0011

Error 18 0.01442 0.00086

Total 29 0.02133

Grand Mean 0.0872 CV 7.21

**Randomized Complete Block ANOVA Table for Glutathione-S-Transferase**

**Source DF SS MS F P**

Replication 2 0.01673 0.00836

Treatment 9 0.04306 0.00478 4.97 0.0019

Error 18 0.01734 0.00096

Total 29 0.07713

Grand Mean 0.0801 CV 5.61

**Randomized Complete Block ANOVA Table for Glutathione reductase**

**Source DF SS MS F P**

Replication 2 0.00525 0.002625

Treatment 9 0.01236 0.004058 2.76 0.0012

Error 18 0.02641 0.001467

Total 29 0.06818

Grand Mean 0.0812 CV 6.41

**Randomized Complete Block ANOVA Table for Guaiacol peroxidase**

**Source DF SS MS F P**

Replication 2 0.06271 0.003135

Treatment 9 0.04373 0.004859 3.078 0.0011

Error 18 0.02842 0.001579

Total 29 0.07842

Grand Mean 0.0414 CV 7.56

**Randomized Complete Block ANOVA Table for Ascorbate peroxidase**

**Source DF SS MS F P**

Replication 2 0.00757 0.003785

Treatment 9 0.07442 0.008269 2.207 0.0010

Error 18 0.06743 0.003746

Total 29 0.14942

Grand Mean 0.0671 CV 5.61

**Randomized Complete Block ANOVA Table for H2O2**

**Source DF SS MS F P**

Replication 2 0.01673 0.00836

Treatment 9 163981 18220.2 1.9E+07 0.0000

Error 18 0.01734 9.633E-04

Total 29 163981

Grand Mean 133.34 CV 0.02

**Randomized Complete Block ANOVA Table for Leaf area**

**Source DF SS MS F P**

Replication 2 0.0167 0.00836

Treatment 9 11.7696 1.30773 1357.50 0.0000

Error 18 0.0173 0.00096

Total 29 11.8036

Grand Mean 4.5154 CV 0.69

**Randomized Complete Block ANOVA Table for Lipid peroxidation (MDA)**

**Source DF SS MS F P**

Replication 2 0.017 0.0084

Treatment 9 744.395 82.7105 85858.7 0.0000

Error 18 0.017 0.0010

Total 29 744.429

Grand Mean 8.7685 CV 0.35

**Randomized Complete Block ANOVA Table for Lipoxygenase**

**Source DF SS MS F P**

Replication 2 0.02 0.00836

Treatment 9 9357.97 1039.77 1079351 0.0000

Error 18 0.02 9.633E-04

Total 29 9358.01

Grand Mean 36.148 CV 0.09

**Randomized Complete Block ANOVA Table for No of roots**

**Source DF SS MS F P**

Replication 2 0.017 0.0084

Treatment 9 145.497 16.1663 16781.6 0.0000

Error 18 0.017 0.0010

Total 29 145.531

Grand Mean 8.0417 CV 0.39

**Genetic components for various traits of maize under salt and drought stress conditions**

| Traits | M.S | G.M | GV | GCV % | PV | PCV % | EV | ECV % | h2bs% | GA% |
| --- | --- | --- | --- | --- | --- | --- | --- | --- | --- | --- |
| Root length | 25.132* | 12.182 | 8.377 | 82.924 | 8.378 | 82.929 | 0.001 | 0.906 | 99.988 | 41.694 |
| Root weight | 0.457* | 0.198 | 0.152 | 87.690 | 0.153 | 87.967 | 0.001 | 6.970 | 99.372 | 345.118 |
| Root-to-shoot length ratio | 0.709* | 0.780 | 0.236 | 55.032 | 0.237 | 55.143 | 0.001 | 3.509 | 99.595 | 109.148 |
| Seed germination %age | 1095.790* | 63.270 | 365.263 | 240.272 | 365.264 | 240.273 | 0.001 | 0.377 | 99.97 | 53.013 |
| shoot length | 7.152* | 13.766 | 2.384 | 41.611 | 2.385 | 41.620 | 0.001 | 0.852 | 99.958 | 19.679 |
| SOD | 10.470* | 4.067 | 3.490 | 92.628 | 3.490 | 92.640 | 0.001 | 1.488 | 99.974 | 80.598 |
| Leaf length | 0.815* | 2.738 | 0.271 | 31.483 | 0.272 | 31.538 | 0.001 | 1.873 | 99.647 | 33.334 |
| Leaf width | 0.157* | 2.277 | 0.052 | 15.096 | 0.053 | 15.235 | 0.001 | 2.053 | 98.183 | 17.399 |
| Shoot weight | 0.173* | 0.282 | 0.057 | 45.164 | 0.058 | 45.539 | 0.001 | 5.837 | 98.357 | 148.080 |
| catalase | 0.003* | 0.080 | 0.001 | 8.703 | 0.001 | 13.532 | 0.001 | 10.362 | 41.364 | 34.708 |
| Gultathione-S- transferase | 0.005* | 0.081 | 0.001 | 12.523 | 0.002 | 16.584 | 0.001 | 10.873 | 57.015 | 58.235 |
| Glutathione reductase | 0.004* | 0.081 | 0.001 | 10.313 | 0.002 | 16.942 | 0.001 | 13.441 | 37.057 | 38.666 |
| Guaiacol peroxidase | 0.005* | 0.041 | 0.001 | 16.251 | 0.003 | 25.407 | 0.002 | 19.529 | 40.913 | 89.657 |
| Ascorbate peroxidase | 0.008* | 0.067 | 0.002 | 14.990 | 0.005 | 27.981 | 0.004 | 23.628 | 28.697 | 54.404 |
| H2O2 | 18220.20* | 133.340 | 6073.400 | 674.894 | 6073.401 | 674.894 | 0.001 | 0.268 | 99.98 | 102.573 |
| Leaf area | 1.308* | 4.515 | 0.436 | 31.059 | 0.437 | 31.093 | 0.001 | 1.458 | 99.780 | 25.624 |
| MDA | 82.711* | 8.769 | 27.570 | 177.319 | 27.571 | 177.322 | 0.001 | 1.068 | 99.996 | 105.090 |
| Lipoxygenase | 1039.770* | 36.148 | 346.590 | 3.096 | 346.591 | 0.515 | 0.001 | 0.515 | 99.97 | 90.386 |
| No of roots | 16.166* | 8.042 | 5.388 | 0.819 | 5.389 | 0.289 | 0.001 | 1.115 | 99.981 | 50.655 |

*=Significant at 5% probability level, Mean Sum of Squares (M.S), Grand mean (G.M), Genotypic variance (GV), Genotypic coefficient of variance (GCV %), Phenotypic variance (PV), Phenotypic coefficient of variance (PCV %), Environmental Variance (EV), Environmental coefficient of variance (ECV %), Broad sense heritability (h^2^bs %), Genetic advance (GA)
